# Positron Emission Tomography (PET) Imaging of Multiple Myeloma in a Post-Treatment Setting

**DOI:** 10.3390/diagnostics11020230

**Published:** 2021-02-03

**Authors:** Giulia Ferrarazzo, Silvia Chiola, Selene Capitanio, Maria Isabella Donegani, Alberto Miceli, Stefano Raffa, Alberto Stefano Tagliafico, Silvia Morbelli, Matteo Bauckneht

**Affiliations:** 1 IRCCS Ospedale Policlinico San Martino, 16132 Genoa, Italy; giulia.ferrarazzo@gmail.com (G.F.); isabella.donegani@gmail.com (M.I.D.); albertomiceli23@gmail.com (A.M.); Stefanoraffa@live.com (S.R.); albertotagliafico@gmail.com (A.S.T.); matteo.bauckneht@gmail.com (M.B.); 2 Nuclear Medicine Unit, Department of Health Sciences, University of Genoa, 16132 Genoa, Italy; 3 Department of Nuclear Medicine, Humanitas Clinical and Research Center-IRCCS, Rozzano, 20089 Milan, Italy; silvia.chiola@gmail.com; 4 Nuclear Medicine, ASST Grande Ospedale Metropolitano Niguarda, Department of Advanced Diagnostic Therapeutic Technologies, 20162 Milan, Italy; selene.capitanio@libero.it; 5 Radiology Unit, Department of Health Sciences, University of Genoa, 16132 Genoa, Italy

**Keywords:** multiple myeloma, positron emission tomography, magnetic resonance imaging, response assessment

## Abstract

2-deoxy-2-[18F]fluoro-D-glucose (FDG) positron emission tomography/computed tomography (FDG PET/CT) has an established clinical value in the diagnosis and initial staging of multiple myeloma (MM). In the last ten years, a vast body of literature has shown that this tool can also be of high relevance for monitoring therapy responses, making it the recommended imaging approach in this field. Starting from the strengths and weaknesses of radiological imaging in MM, the present review aims to analyze FDG PET/CT’s current clinical value focusing on therapy response assessment and objective interpretation criteria for therapy monitoring. Given the potential occurrence of patients with MM showing non-FDG-avid bone disease, new opportunities can be provided by non-FDG PET tracers. Accordingly, the potential role of non-FDG PET tracers in this setting has also been discussed.

## 1. Introduction

Multiple myeloma (MM) is the second most common hematologic malignancy and is associated with the abnormal proliferation of well-differentiated plasma cells [1]. This condition is eventually preceded by an asymptomatic phase (the monoclonal gammopathy of undetermined significance, MGUS), characterized by increased clonal plasma cell levels in the bone marrow without organ involvement [1]. In some cases, an intermediate phase, defined as smoldering multiple myeloma (SMM) is also described [2]. Patients with active MM show a high serum free light chain ratio and plasma cell content in the bone marrow (≥60%) [3]. The evolution from MGUS/SMM to active MM is also associated with the appearance of clinical signs of organ damage including renal insufficiency, hypercalcemia and anemia as well as the presence of bone involvement documented by radiological imaging.

For this reason, imaging technologies have become crucial in many phases of the disease. In particular, low dose computed tomography (CT) and magnetic resonance imaging (MRI) allow the early recognition of osteolytic lesions and the assessment of the bone marrow involvement, respectively. This anatomical description provides relevant information in the earlier phases of the disease. On the other hand, in the last years, a vast body of literature has demonstrated the added value of 2-deoxy-2-[18F]fluoro-D-glucose positron emission tomography/computed tomography (FDG PET/CT) over standard imaging in many phases of the disease including the initial diagnosis and staging [3,4,5,6,7,8], restaging at relapse [9,10,11], prognostic assessment [9,12,13,14,15] and monitoring therapy response. This latter indication has been increasingly studied due to the emerging capability of FDG imaging to detect with high sensitivity the persistence of residual active clonal plasma cells within residual lytic lesions, which are of adverse prognostic significance [16]. Moreover, FDG PET/CT is superior to MRI in the early detection of a response to salvage therapy [17]. These findings supported FDG imaging inclusion in the consensus recommendations by the International Myeloma Working Group (IMWG) [3,18].

On these bases, starting from the strengths and weaknesses of radiological imaging in MM, the present review aims to analyze the current state of the art of FDG PET/CT focusing on the post-treatment setting with a particular interest in the therapy response assessment. Given the potential occurrence of patients with MM showing non-FDG-avid bone disease, new opportunities can be provided by non-FDG PET tracers. Accordingly, the potential role of non-FDG PET tracers in this setting has also been discussed.

## 2. Methods

Aiming to systematically review the available literature on the FDG PET/CT-based response assessment in MM, we combined the following terms (either as text or MeSH) in PubMed, PMC, Scopus, Google Scholar, Embase, Web of Science and the Cochrane library: “multiple myeloma”, “Positron Emission Tomography”, “Fluorodeoxyglucose”, “Response”, “therapy” and “treatment”. The literature analysis was lastly updated in November 2020. No language restriction was applied to the search but only articles in English were reviewed. Similarly, preclinical studies, case reports and case series involving less than five patients were excluded. The systematic literature search returned 375 articles, which were analyzed according to the title and abstract. After the removal of duplicates, 29 articles were considered and fully read (Table 1). This approach led to the exclusion of 346 articles. Aiming to contextualize the above-mentioned topic, a further literature search focusing on the remaining clinical applications of FDG PET/CT in MM, on the complementary role of MRI and on non-FDG PET tracers was also performed through the same databases. Due to the extensive existing literature about these topics, these articles were not systematically reviewed. Therefore, the corresponding sections represent a narrative description of the clinical background for FDG PET/CT imaging therapy monitoring in this field. 

## 3. Value and Limits of MRI in Multiple Myeloma in the Post-Treatment Setting

**Figure 1:**
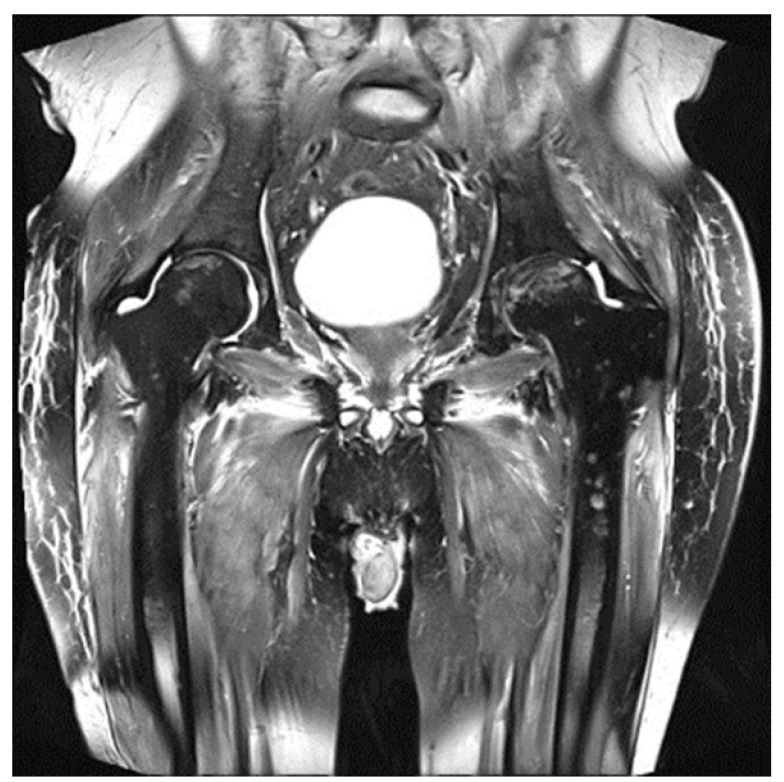
An emblematic case of bilateral femoral head osteonecrosis in a post-transplanted multiple myeloma (MM) patient. In the femoral head osteonecrosis is a double line sign with a low signal intensity and outer rim and high signal intensity inner line as demonstrated on the coronal T2-weighted image.

Since 2014, the IMWG has included MRI instead of standard radiography in the MM diagnostic criteria [3,18]. Thanks to its high sensitivity and the absence of radiation exposure, this tool can be used for diagnostic and prognostic purposes in various phases of the disease [42,43]. However, due to its functional nature, besides the mere anatomic description of the MM-related bone subversion, MRI can also evaluate the response of therapy. Through the injection of a gadolinium-based contrast medium, it allows the estimation of neoangiogenesis. The resulting time-intensity curve temporal variation can be quantitatively analyzed, allowing the measurement of the decrease in MM perfusion [44], which correlates with a biochemical response [45,46]. Similarly, the quantification of active tumor load as displayed by diffusion weighted sequences (DWI, which represent the diffusion of water molecules) appears to differentiate between treatment responders and non-responders [47,48], allowing the prediction of response to induction and consolidation chemotherapy [49]. Finally, MRI can display the occurrence of several treatment-related side effects including the osteonecrosis of the femoral head (Figure 1).

However, since the role of MRI in MM is still expanding, several further functional data can be added to the standard morphological parameters. This rapidly evolving scenario implies the extreme variability in the choice of imaging protocols and the use of contrast agents by the available studies, representing the current major limitation of this tool in the post-treatment setting [50,51]. On these bases, several efforts have been dedicated to the standardization and the decrease of variations in the acquisition, interpretation and reporting of MRI, allowing better response assessments that has led to the Myeloma Response Assessment and Diagnosis System (MY-RADS) [50]. MY-RADS criteria are designed to provide a comprehensive characterization of the myeloma state at diagnosis, at the start of treatment, after therapy and during follow-up. However, it requires validation within clinical trials including assessments of reproducibility, correlations with the biochemical response, skeletal events, progression-free survival (PFS) and overall survival (OS).

A further matter of debate is the adequate timing of MRI imaging in the post-treatment setting. Indeed, a decrease in the apparent diffusion coefficient (ADC) following therapy can be considered an indirect index of response as it indicates the degree of water movement within extracellular and intracellular space (proxies of tissue cell density), which typically decreases during the transition from active disease to remission [51]. However, the direction of the ADC change can be profoundly influenced by imaging timing. Messiou et al. showed a transient increase in ADC values soon after therapy, presumably related to plasma cell death and the resulting increased extracellular space [51]. Therefore, to improve the appropriateness of MRI image interpretations, it should always coincide with clinical routines where serum and marrow assessments are performed [50].

## 4. FDG PET/CT Images Interpretation in Therapy Monitoring of Multiple Myeloma

The functional nature of FDG imaging may also allow the assessment of treatment response in MM. Indeed, while morphologic changes of lytic lesions remain relatively stable in time (scarcely displaying treatment efficacy), metabolic changes related to treatment occur in a relatively short time and can be easily measured and monitored.

Regarding metabolic activity, MM includes a heterogeneous spectrum ranging from low to extremely high FDG-avid disease [52,53]. Consequently, both false-positive and negative results may occur when monitoring the FDG imaging response in MM [53]. Sources of false-positive results include many conditions such as inflammation, recent bone fractures, post-surgical or vertebroplasty sites, bone remodeling, the presence of orthopedic devices with consequent significant artifacts on CT images, infection, diffuse bone marrow uptake such as under specific treatments (chemotherapy, radiotherapy, use of growth factors) or in other clinical states associated with a hot background in the bone (i.e., an anemic condition or the administration of erythropoietin) [16]. On the other hand, PET sensibility can be hampered by elevated glycemic levels, high-dose steroid therapy, low hexokinase-2 expression [52,53] or the presence of pure lytic lesions characterized by a low FDG uptake or an early PET-positive lesion without a correspondent osteolytic area. Furthermore, the spatial resolution of PET imaging could be insufficient to detect the typical salt and pepper Bone Marrow (BM) infiltration or to identify the occurrence of small lytic lesions in specific anatomic districts (such as the skull with the close brain physiological activity), generating possible misinterpretations [54].

The identification of standardized imaging criteria is thus of pivotal importance to estimate MM’s extent and metabolic activity in the everyday clinical use of FDG PET/CT, particularly in the post-treatment setting. On these bases, in the last years several studies have tried to identify clinically valuable PET derived indexes and to harmonize PET scan interpretation, thus overcoming the limited reproducibility of the several previous clinical trials in different clinical settings [16,32,54]. During PET reporting, image interpretation is generally based on a pure visual assessment, semiquantification or both methods.

The visual inspection of FDG PET/CT images remains undoubtedly the first step because it is free from technical artifacts and allows the harmonization of PET reporting [55]. Nevertheless, Standardized Uptake Value (SUV) calculation allows a more standardized estimation of metabolic activity particularly in the response assessment to therapy. Within the same PET center, the measure of SUV can thus eliminate inter-observer variations. However, when a given semiquantitative positivity cut-off is set, different image reconstruction algorithms could be adopted for the different scanners. This discrepancy, especially in the absence of international scanner calibration, contributes to an increase in SUVmax variability, hampering image interpretation and worsening the reproducibility of the results particularly in borderlines cases. For these reasons, a few studies have proposed using this parameter in relation to a background reference region such as physiologic bone marrow uptake in lumbar vertebrae, a mediastinal blood pool or physiological liver uptake [55]. Aside from the use of normalized semiquantitative indexes, additional PET derived volumetric parameters such as the metabolic tumor volume (MTV) and total lesion glycolysis (TLG) have been proposed as well. A few studies have analyzed the variation of these parameters after therapy as an index of treatment response [9,56].

After the initial tentative research by Mesguich et al. [57], who proposed a series of general indications for FDG PET/CT interpretation in patients with MM at different stages of the disease, an Italian group proposed visual descriptive criteria termed Italian Myeloma Criteria for PET Use (IMPeTUs) [30], specifically dedicated to the evaluation of the end of therapy PET compared with the baseline. These criteria were derived from the Deauville criteria used for the FDG-PET response assessment in Hodgkin lymphoma [58]. Furthermore, even if they were aware of the limitations derived from the use of different PET scanners with a consequent variation in SUV measurement, the authors also considered a semiquantitative analysis (lesion-to-background SUV ratio) in case of equivocal results. Despite a lower concordance in the recognition of skull lesions and of lesions still active after therapy as common pitfalls in MM, they obtained good results in terms of reproducibility among reviewers, at least in a cohort of 22 patients. These results were subsequently reproduced by the same group in a larger cohort [35]. Of note, when enlarging the sample size, a higher agreement was reached for lesions with a score 4 involving bone marrow and soft tissues in the post-therapy assessment.

IMPeTUs descriptive criteria proved to be highly reproducible, relatively easily applicable in clinical practice and recognized as the first step towards the harmonization of PET interpretation in MM patients. However, the complementary role of PET and morphological imaging is another aspect that must be considered in imaging reporting and it is even more relevant in the post-therapy setting in which functional changes usually precede morphological ones. Accordingly, IMPeTUs criteria require the reporting of the degree of the bone marrow involvement by MM and the inclusion of a number of additional characteristics of the disease to provide a complete picture of the pathological involvement. These further data include the number and site of hypermetabolic foci, the coexistence of lytic lesions and the presence of extra or para-medullary involvement as well as the presence of fractures.

As a further source of validation, IMPeTUs criteria were also used to define a posteriori positivity cut-off using patients’ follow-up data in a large population to identify subjects with active disease, especially in the post-therapy setting. In particular, the presence of a score ≥4 in the bone with either a focal or a diffuse pattern predicted a worse outcome [22].

**Figure 2:**
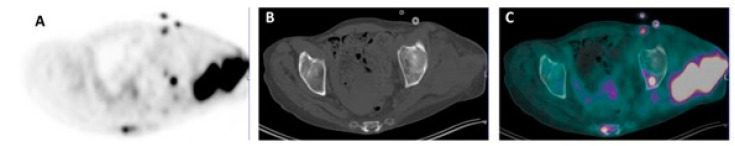
A representative example of hot spots with no underlying lytic lesions. (**A**) positron emission tomography (PET) axial cut, (**B**) computed tomography (CT) axial cut, (**C**) fused images axial cut of two focal uptakes localized on the left acetabulum and coccygeal bone, respectively. Extranodal involvement is also evident on the left side. In this case, IMPeTUs criteria would have been scored as follows: BM2 (normal bone marrow), F2 (two focal hot lesions) with DS4Sp (spinal) and ExP (extraspinal in the left acetabulum), EMmus (extramedullary muscle).

**Figure 3:**
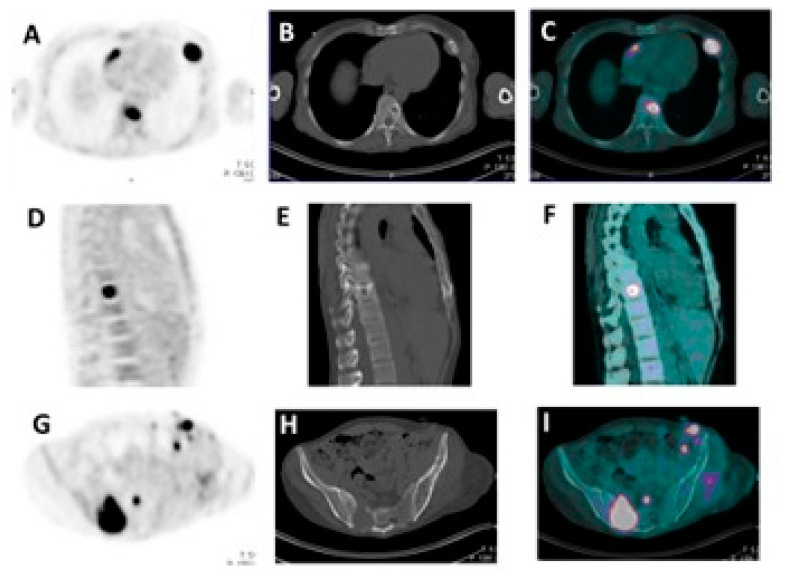
A representative example of hot spots with lytic lesions underlying (**A**,**G**) PET axial cut; (**B**,**H**) CT axial cut; (**C**,**I**) fused images axial; (**D**) PET sagittal cut; (**E**) CT sagittal cut; (**F**) fused images sagittal cut showing active disease in the spine (**A**–**D**), sacrum (**G**–**I**) and in the seventh rib on the left. (**A**–**C**). A pelvic subcutaneous lesion is also evident (**G**–**I**). In this case, IMPeTUs criteria would have been scored as BM4 (increased bone marrow uptake), F2 (three focal hot lesions) with DS4Sp (spinal) and ExP (extraspinal in the rib), EMsk (extramedullary skin).

The strength of these criteria was also confirmed in recent work by Shengming et al. [59]. IMPeTUs resulted in being significantly more accurate concerning the Durie–Salmon and the Revised International classification systems. Indeed, a profound knowledge of MM’s aspects allows a better comprehension of which imaging finding must be underlined for its clinical relevance or its prognostic impact, which instead is collateral or intrinsically equivocal. In this way, it would be possible to draw up a correctly structured image report with a clinical impact and, at the same time, potential scientific significance. Reaching a good inter-observer reproducibility in interpreting the results through a well-accepted classification system such as IMPeTUs could improve the extrapolation of prognostic data from PET images and patients’ risk stratification guiding clinicians in the identification of the best personalized treatment for each patient. Examples of the usage of IMPeTUs criteria for PET findings are given in Figure 2 and Figure 3.

More recently, a joint analysis of a subgroup of newly diagnosed transplantation-eligible patients with MM enrolled in two independent European randomized phase III trials (IFM/DFCI2009 and EMN02/HO95) was performed by Zamagni et al. using the same approach [39]. The analysis of enrolled patients showed that focal or diffuse bone marrow FDG uptake lower than the liver background after therapy was an independent predictor for improved PFS and OS. The authors consequently proposed this criterion as the gold standard for a PET complete metabolic response definition for patients with MM, further confirming the Deauville score’s value in patients with MM.

## 5. Diagnostic and Prognostic Value of FDG PET/CT in the Response Assessment in Multiple Myeloma

As detailed in Table 1, several studies assessed the diagnostic accuracy of FDG PET/CT in the response assessment. Derlin et al. retrospectively compared FDG imaging and whole-body MRI to determine the remission status after BM Transplant (BMT) [25], showing that the former approach was more specific (85.7% vs. 38.1%) for the identification of the remission status due to the lower incidence of false-positive findings. Superimposable results were prospectively observed by Basha et al. [34]. However, the addition of functional parameters (such as ADC) to MRI may favorably impact the specificity of this tool, improving its performance in the response assessment [40]. On the other hand, FDG PET/CT sensitivity may not be uniformly high as it can be lower to the pretreatment phase due to the functional bone marrow FDG uptake, which may reduce the signal to noise ratio [11]. Furthermore, it may depend on the disease category according to the Uniform Response Criteria for myeloma [11]. However, emerging data has shown that dual time point imaging might favorably impact this limitation. Indeed, in a small prospective study by Zirakchian Zadeh et al. [41] observed that the bone marrow FDG uptake between two acquisition time points (at 1- and 3-h post-injection) increased significantly in patients with a poor response to treatment but not in patients that achieved a complete response. This finding might potentially improve FDG PET/CT sensitivity in this differential diagnosis. However, larger studies are needed to confirm this initial evidence.

In addition to its diagnostic value, several studies have underlined that the prognostic significance of FDG PET/CT in the post-treatment setting could guide the subsequent clinical management in MM (Table 1). Bartel et al. [16] performed a subanalysis of the Total Therapy 3 Trial [60], showing that candidates to BMT who did not achieve a complete FDG suppression after induction chemotherapy were characterized by an inferior long-term prognosis. The same results were confirmed by other studies [16,23,27,33,36], supporting the use of serial FDG PET/CT to individualize patient therapy and (eventually) to rapidly move to alternative therapies in the presence of persistent PET positivity before BMT. Of note, the predictive value of the pre-BMT persistent FDG positivity largely overcame one persistent MRI abnormality [16]. The persistence of metabolically active disease (particularly in the extramedullary sites) predicts unfavorable outcomes in the post-BMT setting, being associated with higher relapse rates and shorter PFS and OS [26,28,32,33,37].

## 6. Unmet Needs and Open Issues

Despite the increasing FDG PET/CT role in MM, several issues remain unsolved, particularly in response assessment. First, the exact timing for the PET/CT treatment monitoring is currently lacking as a high heterogeneity in the time of imaging was observed in the analyzed studies. In most cases, post-therapy FDG PET/CT imaging was performed after at least three months from chemotherapy initiation [5,6,12,19,21,25,28,29,37,38]. A few studies [12,23,24] showed the potential utility of a very early response assessment (after one to three cycles of chemotherapy) in predicting the subsequent clinical outcome. In three studies, the post-treatment evaluation timing was pre-BMT setting (after induction chemotherapy) [16,27,39], while a few studies focused on the long-term post-BMT set [11,25].

In addition to its timing, the FDG PET/CT’s exact impact on the subsequent clinical management still needs to be defined. Indeed, there is no study supporting the usefulness of an early treatment change based on the FDG PET/CT result, mainly when it happens in the absence of new osteolytic lesions.

Third, the optimal definition of a metabolic response after treatment is still lacking, hampering meaningful comparisons between the analyzed studies. However, as detailed above, introducing highly reproducible Deauville-derived interpretation criteria represents a promising step toward harmonization in this field.

As a final remark, it should also be noted that both false-positive and negative results may occur when monitoring the response using FDG imaging in MM. Consequently, it is reasonable that in specific clinical situations, approaches other than FDG-PET/CT may be more appropriate to evaluate MM’s response to therapy.

## 7. Non-FDG PET Tracers in the Post-Treatment Setting of Multiple Myeloma

In the last years, several new tracers beyond FDG have also been tested in patients with MM (Table 2).

One of the first non-FDG tracers used in this setting is ^11^C-methionine (MET). MET is able to reflect the synthetic protein turnover by malignant cells and its uptake is not influenced by non-disease related determinants of bone marrow tracer uptakes such as anemia or systemic inflammation. This feature results in a good sensitivity concerning FDG in describing the degree of bone infiltration in the staging phase [61,62,63], even when low monoclonal protein-producing myelomas such as IgD, IgE and non-secretory types are studied [64]. The higher adherence of MET uptake (compared with FDG uptake) to the proliferative activity of MM resulted also in a higher sensitivity in the detection of minimal residual disease in a young patient with an unusual extramedullary (vulvar) presentation of recurrent MM [65]. On these bases, MET-PET/CT was also tested in the response assessment to therapy. Luckerath et al. [66] compared FDG- and MET-PET/CT in the monitoring of responses with anti-myeloma therapy and outcome prediction in MM’s mouse model. The authors showed that MET was more sensitive than FDG in the very early response assessment as CD138 expression reduction on the MM cellular surface was better correlated with MET uptake than FDG [67]. In turn, this variation in tumor biology correlated with survival [66]. Whether confirmed in humans, MET-PET imaging would establish a novel approach for treatment individualization, allowing for therapy initiation and adjustments earlier than any other existing method, even outperforming analysis of free light chains [66].

Other tracers such as NaF, choline, acetate, FLT and PSMA already included in the evaluation of cancer patients in different clinical settings have also been proposed in the field of MM [31,38,68,69,70,71,72]. Preliminary evidence in the preclinical setting and small groups of patients are also available with other tracers such as [^68^Ga]Pentixafor [73], 18F-Fludarabine [74] and a radiolabeled anti-CD138 murine antibody [75]. However, these studies mostly focused on the PET detection of MM lesions rather than the response to therapy or residual disease detection.

## 8. Conclusions

In conclusion, FDG PET/CT has an established clinical value in the initial phase of MM. However, in the last ten years, emerging data have shown that this tool could be of a high value for monitoring the therapy response making FDG PET/CT the recommended imaging approach in this field. This has raised the need for standardized imaging evaluation criteria to uniformly estimate the metabolic response to treatment in clinical trials and everyday clinical practice. Non-FDG PET tracers may explore MM’s other biological features, thus further improving the response assessment of plasma cell disorders.

## Figures and Tables

**Table 1:** Available studies on FDG PET/CT in therapy monitoring multiple myeloma.

Ref	First Author, Year	Country	Number of Patients	Population	Study Design	Administered Therapy	Timepoint	Follow-Up Duration	Images Evaluation	Endpoint/Gold Standard	Major Findings
[19]	Jadvar, H., 2002	USA	6	MM	PCS	CTx + auto-BMT	Baseline and 3 months after therapy	n.a.	Qualitative	CO	Clinical outcome following treatment administration is paralleled by FDG uptake changes
[20]	Mileshkin, L., 2004	Australia	69	MM	RS	RT	Baseline	n.a.	Qualitative	n.a.	FDG PET allows the evaluation of the presence of ongoing disease activity in previously irradiated sites remaining abnormal at skeletal imaging after treatment
[5]	Bredella, M.A., 2005	USA	9/13	MM	RS	CTx, RT, surgery, or BMT	Baseline and 3 months after therapy	n.a.	Qualitative, semiquantitative (SUVmax, SUVmean)	CO	FDG PET was helpful in describing post-therapeutic changes. However, there was one false-positive FDG PET result in a patient who had undergone RT three weeks before PET
[6]	Zamagni, E., 2007	Italy	23/46	MM	PCS	Auto-BMT	Baseline and 3 months after therapy	n.a.	Qualitative, semiquantitative (SUVmax)	M protein concentration, MRI, CO	FDG PET/CT overcame MRI in the evaluation of the response following BMT
[21]	Kim, P.J., 2008	USA, Australia	11/17	Plasmacytoma	RS	RT	Baseline and 2–4 months after therapy	4–96 months	Qualitative, semiquantitative (SUVmax)	CO	Restaging PET scans after RT successfully assessed the response to treatment in all. Of note, two patients showed late responses
[16]	Bartel, T.B., 2009	USA	239	MM	PCS	Total Therapy 3 scheme [22]	Baseline, before each BMT, before consolidation and maintenance and semiannually thereafter	43 months (median)	Qualitative, semiquantitative (SUVmax)	PFS and OS	MM survival can be improved by altering treatment in patients in whom FDG suppression is not achieved after induction CTx
[23]	Dimitrakopoulou-Strauss, A., 2009	Germany	19	MM	PCS	CTx	Baseline and 2–5 weeks after therapy	0–64.1 months	Qualitative, semiquantitative and dynamic imaging parameters calculated according to Patlak model	PFS	Early FDG kinetics studies (after 1 cycle of CTx) successfully predict the subsequent PFS in MM
[24]	Sager, S., 2011	Turkey	10/42	MM, plasmacytoma	RS	n.a.	Baseline and 3 weeks after therapy	At least 6 months	Qualitative, semiquantitative (SUV)	CO	FDG PET allows the evaluation of the presence of ongoing disease activity in previously irradiated sites remaining abnormal at skeletal imaging after treatment
[12]	Zamagni, E., 2011	Italy	192	MM	PCS	Induction CTx and double auto-BMT	Baseline, within 10 days after induction therapy, 3 months after auto-BMT, during follow-up and at the time of relapse	42 months (median)	Qualitative, semiquantitative (SUVmax)	PFS and OS	The persistence of hypermetabolic lesions after induction CTx is an early predictor for shorter PFS. Three months after auto-BMT, a negative FDG PET/CT is associated with a more favorable 4-year rate of PFS and OS with respect to PET-positive
[11]	Derlin, T., 2012	Germany	99	MM	RS	Auto or allo-BMT	post-BMT setting (median interval 33.9 ± 31.5 months, range 1.2–143.1)	n.a.	Qualitative, semiquantitative (SUVmax)	CO	Post-BMT FDG PET/CT contributes to the restaging but has a substantially lower sensitivity for this purpose compared with the pretreatment setting
[25]	Derlin, T., 2013	Germany	31	MM	RS	Autologus or allogenic BMT	post-BMT setting (median interval 37.4 ± 38.1 months, range 2.4–143.1).	n.a.	Qualitative, semiquantitative (SUVmax)	Clinical remission status (Uniform Response Criteria)	PET/CT is more accurate than MRI for the determination of the remission status after BMT
[26]	Nanni, C., 2013	Italy	107	MM	PCS	Auto-BMT	Baseline, after 3 months from BMT and every 6–12 months during follow-up	41 months (mean)	Qualitative, semiquantitative (SUVmax)	DFS, TTR	In the post-BMT setting, a negative FDG PET/CT predicts favorable DFR and TTR while metabolically active disease persistency is correlated with shorter TTR
[27]	Usmani, S.Z., 2013	USA	302	MM	PCS	Total Therapy 3 scheme [22]	Baseline, at day 7 from induction and before the first BMT	6.8 years (TTR 3A) and 4.3 years (TTR 3B)	Qualitative, semiquantitative (SUVmax)	CRD, PFS, OS	The presence of more than 3 PET focal lesions after day 7 first cycle of induction CTx predicts inferior PFS and OS
[28]	Patriarca, F., 2015	Italy	59/67	MM	RS	Allo-BMT	Baseline, after 6 months from BMT and every 12 months in the follow-up	6 months (range 1–122)	Qualitative, semiquantitative (SUVmax)	PFS, OS	The persistence of extramedullary disease and a failure to obtain a metabolic response after allo-BMT are associated with shorter PFS and OS
[15]	Zamagni, E., 2015	Italy	282	MM	RS	CTx and/or BMT	Baseline, after 3 months from the first line of therapy and every 12–18 months during post-treatment follow-up	67 months (median)	Qualitative, semiquantitative (SUVmax)	PFS, OS	The failure to achieve a compete metabolic response after the first-line treatment predicts lower PFS and OS
[29]	Li, Y., 2017	China	67/98	MM	PCS	CTx and BMT	Baseline, after BMT	16.63 months (range 4.97–33.33)	Qualitative, semiquantitative (SUVmax, T/Mmax)	DFS, OS	T/Mmax overcomes SUVmax for the evaluation of the treatment response
[30]	Nanni, C., 2016	Italy	17	MM	PCS	CTx and BMT	Baseline, after induction, end of therapy	n.a.	Qualitative, semiquantitative (SUVmax), IMPeTUs criteria	n.a.	Response assessment by means of IMPeTUs criteria is feasible in the clinical practice
[31]	Sachpekidis, C., 2017	Germany	29/34	MM	PCS	CTx and BMT	Baseline, after 3 months from therapy	15–52 months	Qualitative, semiquantitative (SUVmax), quantitative	PFS, OS	18F-NaF PET/CT does not add significantly to 18F-FDG PET/CT in the treatment response evaluation of MM
[32]	Moreau, P., 2017	France	134	MM	PCS	CTx +/− BMT	Baseline, after three cycles of CTx and before maintenance	30 months	Qualitative, semiquantitative (SUVmax)	PFS, OS	FDG PET/CT normalization before maintenance is associated with better PFS and OS
[33]	Stolzenburg, A., 2018	Germany, USA	52	MM	RS	Allo-BMT	Before and after BMT (91 ± 50 days after)	62.3 months (range 29–124)	Qualitative, semiquantitative (SUVmax)	PFS, OS	FDG PET/CT negativity prior to or following allo-BMT is a favorable prognostic factor for PFS and OS
[34]	Basha, M.A., 2018	Egypt	22/56	MM	PCS	CTx, RT or BMT	Baseline, after 6 months from therapy	9 months	Qualitative, semiquantitative (SUVmax)	CO, bone marrow biopsy	FDG PET/CT is more specific than whole-body MRI in detecting residual disease in treated patients
[35]	Nanni, C., 2018	Italy	86	MM	PCS	CTx and BMT	Baseline, after induction, end of therapy	n.a.	Qualitative, semiquantitative (SUVmax), IMPeTUs criteria	n.a.	Response assessment by means of IMPeTUs criteria is highly reproducible in the clinical practice
[22]	Zamagni, E., 2018	International multicentric	236	MM	PCS	CTx and BMT	Baseline, prior to the start of maintenance	62.9 months (median)	Qualitative, semiquantitative (SUVmax), IMPeTUs criteria	PFS, OS	Reduction of the focal lesion and bone marrow FDG uptake to a lower degree than the liver after therapy independently predicts PFS and OS
[36]	Bailly, C., 2018	France	71	MM	PCS	CTx	Baseline and after three cycles of CTx	21.5 months (median)	Qualitative, semiquantitative (SUVmax, ΔSUVmax), IMPeTUs criteria	BR, PFS	Early FDG PET/CT response assessment (interim) predicts the long-term CO
[37]	Ripani, D., 2019	Italy	28	MM	RS	CTx and BMT	Baseline and 4.8 ± 1.5 months after treatment completion	48.2 ± 9.8 months (mean)	Qualitative and semiquantitative (SUVmax, SUVmean, SUVpeak, MTVsum, TLGsum, rPET, qPET)	TMP	Semiquantitative normalized FDG PET-CT parameters outperform non-normalized indexes in the prediction of persistent response to treatment
[38]	Nakuz, T.S., 2019	Austria	7	MM	PCS	CTx, RT, BMT	Baseline, at 10 and 17 months from therapy beginning	45 months (median)	Qualitative, semiquantitative (SUVmax, SUVpeak, SUVmean)	BR	FDG PET/CT overcomes NaF PET/CT in the description of persistent treatment-related metabolic changes
[39]	Zamagni, E., 2020	International multicentric	228	MM	PCS	CTx +/− BMT	Baseline, after induction CTx, before maintenance	62.9 months (median)	Qualitative, semiquantitative (SUVmax), IMPeTUs criteria	PFS, OS	After therapy focal lesions and bone marrow FDG uptake lower than the liver background independently predicts PFS and OS
[40]	Paternain, A., 2020	Spain	27	MM	PCS	CTx +/− BMT	Baseline, after treatment (median 102 days after)	n.a.	Qualitative, semiquantitative (SUVmax), IMPeTUs criteria	IMWG response criteria	DWI-MRI and ADC correlates with FDG PET/CT and the IMWG response criteria
[41]	Zirakchian Zadeh, M., 2020	USA	36	MM	PCS	CTx +/− auto-BMT	At baseline and after therapy	n.a.	Semiquantitative: global SUVmean (GSUVmean)	IMWG response criteria	Dual time point (1 and 3 h post-injection) FDG PET/CT imaging may improve the treatment response assessment

ADC: Apparent Diffusion Coefficient; BMT: Bone marrow transplant; BR: Biochemical response; CO: Clinical Outcome; CRD: Complete response duration; CTx: Chemotherapy; DFS: Disease-free survival; DWI: Diffusion weighted imaging; IMWG: International Myeloma Working Group; MM: Multiple myeloma; MRI: Magnetic resonance imaging; MTV: Metabolic Tumor Volume; OS: Overall Survival; PCS: Prospective; PFS: Progression Free Survival; qPET: Lesion SUVpeak/liver SUVmean; rPET: Lesion SUVmax/liver SUVmax; RS: Retrospective; RT: Radiotherapy; SUV: Standardized Uptake Value; T/Mmax: Ratio of SUVmax in lesions to SUVmax in the mediastinum; TLG: Total Lesion Glycolysis; TMP: Time to Metabolic Progression; TTR: Time to Relapse.

**Table 2:** Non-2-deoxy-2-[18F]fluoro-D-glucose (FDG) PET tracers in the post-treatment setting of multiple myeloma.

Ref	First Author, Year	Country	Number of Patients	Population	Tracer	Study Design	Administered Therapy	Timepoint	Follow-Up Duration	Images Evaluation	Endpoint/Gold Standard	Major Findings
[70]	Lin, C., 2014	Taiwan	13/15	MM	ACT	PCS	Induction therapy	Baseline and post-induction therapy	4 months	Qualitative, semiquantitative (SUVmax)	CO, FDG PET/CT, MRI	Acetate PET/CT showed a higher detection rate for myeloma lesions at diagnosis than using 18F-FDG and may be valuable for response evaluation
[65]	Caldarella, C., 2017	Italy	1	Extramedullary relapse of MM	MET	RS	CTx	Baseline and post-CTx	n.a.	Qualitative	FDG PET/CT	Additional role of MET PET/CT in comparison with FDG PET/CT in depicting possible residual disease after treatment in extramedullary vulvar relapse of MM in a young patient
[64]	Imataki, O., 2017	Japan	1	Serologically less active myeloma	MET	RS	CTx	Baseline and after CTx	n.a.	Qualitative	FDG PET/CT	MET-PET/CT is more sensitive than FDG-PET in patients with serologically less active myeloma
[73]	Lapa, C., 2017	Germany	35	MM	Pentixafor	RS	CTx + 28/35 auto-BMT	After CTx + 28/35 auto-BMT	n.a.	Qualitative, semiquantitative (SUVmax, SUVmean)	OS, PFS, FDG PET/CT	Pentixafor-PET/CT provides further evidence that CXCR4 expression frequently occurs in advanced MM, representing a negative prognostic factor and a potential target for specific treatment
[62]	Lapa, C., 2017	Germany and Spain	78	Plasmacytoma, SMM, MM	MET	PCS	Baseline or CTx/RT/auto-BMT	Baseline or after therapy	n.a.	Qualitative, semiquantitative (SUVmax)	Histologic plasma cell infiltration, FDG PET/CT	MET PET/CT shows higher sensitivity in comparison with standard FDG to detect intra and extramedullary MM
[31]	Sachpekidis, C., 2017	Germany	34	MM	NaF	PCS	CTx + auto BMT	Baseline and after therapy	15–52 months	Qualitative, semiquantitative (SUVmax, SUVmean)	OS, PFS, FDG PET/CT	NaF PET/CT did not aid significantly in treatment response assessment of MM patients, at least in an early phase
[72]	Sasikumar, A., 2017	India	1	Plasmacytoma	PSMA	RS	Baseline	baseline	n.a.	Qualitative	FDG PET/CT	PSMA PET/CT allows the imaging of MM
[69]	Sachpekidis, C., 2018	Germany	12	MM, SMM	FLT	PCS	Baseline or CTx + auto-BMT	11/12 baseline	n.a.	Quantitative, semiquantitative (SUVmax, SUVmean), quantitative	FDG PET/CT	FLT does not seem suitable as a single tracer in MM diagnostics
[68]	Lapa, C., 2019	Germany	19	MM, plasmacytoma	MET and choline	RS	Baseline	Baseline	n.a.	Qualitative, semiquantitative (SUVmax, SUVmean)	BM biopsy	MET PET/CT could be more sensitive than choline PET/CT for the detection of active MM lesions
[29]	Nakuz, T.S., 2019	Austria	7	MM	NaF	RS	Baseline	Baseline and after therapy	7–22 months	Qualitative, semiquantitative (SUVmax, SUVmean)	CO, BM infiltration, FDG PET/CT	NaF PET/CT as a marker of bone mineralization was shown to be significantly decreased after first-line therapy
[63]	Morales-Lozano, M.I., 2020	Germany	22	MM	MET	RS	Baseline	Baseline	n.a.	Qualitative, semiquantitative (SUVmax, SUVmean, SUVpeak, MTV), quantitative	CO, R-ISS, M protein concentration, FDG PET/CT	MET PET/CT is a more sensitive marker for the assessment of myeloma tumor burden than 18F-FDG
